# Molecular determinants of sulfadoxine-pyrimethamine resistance in *Plasmodium falciparum* in Nigeria and the regional emergence of *dhps* 431V

**DOI:** 10.1016/j.ijpddr.2016.08.004

**Published:** 2016-09-29

**Authors:** Mary C. Oguike, Catherine O. Falade, Elvis Shu, Izehiuwa G. Enato, Ismaila Watila, Ebenezer S. Baba, Jane Bruce, Jayne Webster, Prudence Hamade, Sylvia Meek, Daniel Chandramohan, Colin J. Sutherland, David Warhurst, Cally Roper

**Affiliations:** a Department of Immunology and Infection, Faculty of Infectious and Tropical Diseases, London School of Hygiene and Tropical Medicine, London, United Kingdom; b Department of Pharmacology and Therapeutics, College of Medicine, University of Ibadan, Ibadan, Nigeria; c Department of Pharmacology and Therapeutics, College of Medicine, University of Nigeria, Enugu Campus, Enugu, Nigeria; d Department of Child Health, University of Benin Teaching Hospital, Benin City, Nigeria; e Department of Paediatrics, Specialist Hospital Maiduguri, Borno State, Nigeria; f Malaria Consortium, Regional Office for Africa, Kampala, Uganda; g Department of Disease Control, Faculty of Infectious and Tropical Diseases, London School of Hygiene and Tropical Medicine, London, United Kingdom; h Malaria Consortium, London, United Kingdom; i Department of Pathogen Molecular Biology, Faculty of Infectious and Tropical Diseases, London School of Hygiene and Tropical Medicine, London, United Kingdom

**Keywords:** Sulfadoxine-pyrimethamine, *dhps*, *dhfr*, mutations, Nigeria

## Abstract

There are few published reports of mutations in dihydropteroate synthetase (*dhps)* and dihydrofolate reductase (*dhfr)* genes in *P. falciparum* populations in Nigeria, but one previous study has recorded a novel *dhps* mutation at codon 431 among infections imported to the United Kingdom from Nigeria. To assess how widespread this mutation is among parasites in different parts of the country and consequently fill the gap in sulfadoxine-pyrimethamine (SP) resistance data in Nigeria, we retrospectively analysed 1000 filter paper blood spots collected in surveys of pregnant women and children with uncomplicated falciparum malaria between 2003 and 2015 from four sites in the south and north.

Genomic DNA was extracted from filter paper blood spots and placental impressions. Point mutations at codons 16, 50, 51, 59, 108, 140 and 164 of the *dhfr* gene and codons 431, 436, 437, 540, 581 and 613 of the *dhps* gene were evaluated by nested PCR amplification followed by direct sequencing.

The distribution of the *dhps*-431V mutation was widespread throughout Nigeria with the highest prevalence in Enugu (46%). In Ibadan where we had sequential sampling, its prevalence increased from 0% to 6.5% between 2003 and 2008. Although there were various combinations of *dhps* mutations with 431V, the combination 431V + 436A + 437G+581G+613S was the most common.

All these observations support the view that *dhps*-431V is on the increase. In addition, *P. falciparum* DHPS crystal structure modelling shows that the change from Isoleucine to Valine (*dhps*-431V) could alter the effects of both S436A/F and A437G, which closely follow the 2nd β-strand. Consequently, it is now a research priority to assess the implications of *dhps-*VAGKGS mutant haplotype on continuing use of SP in seasonal malaria chemoprevention (SMC) and intermittent preventive treatment in pregnancy (IPTp). Our data also provides surveillance data for SP resistance markers in Nigeria between 2003 and 2015.

## Highlights

• We present data on *dhps* and *dhfr* mutations in *P. falciparum* populations in Nigeria.
• Increased prevalence of I431V mutation was seen between 2003 and 2015 from 0 to 36%.
• The 431V + 436A + 437G + 581G + 613S was the most common with *dhps*-431V mutation.
• Crystal structure modelling of *Pf* DHPS shows that 431Vcould alter S436A and A437G.

## Graphical abstract

**Figure:**
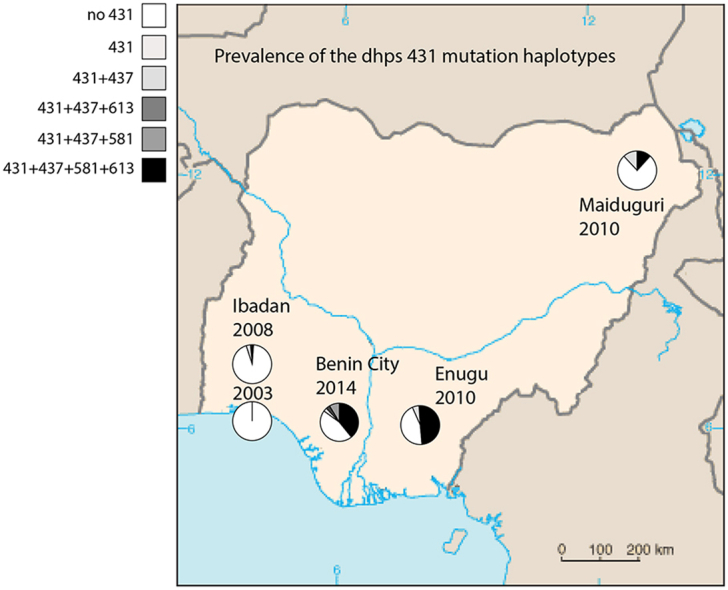

## 1 Introduction

Malaria is a major public health challenge in sub-Saharan Africa. In 2015, there was an estimated 214 million cases and 438,000 deaths due to malaria globally with Nigeria accounting for 25% of these (WHO, 2015). Malaria poses health risks to both neonate and mother during pregnancy. It leads to low birth-weight, placental malaria, severe maternal anaemia (especially in primigravidae), and perinatal mortality (Shulman et al., 1999, Menendez et al., 2010, Oyibo and Agomo, 2011). Pregnant women are usually at higher risk of malaria infection than their non-pregnant counterparts due to temporary depression of immunity during foetal development (Menendez, 1995). The World Health Organization (WHO) has recommended intermittent preventive treatment during pregnancy (IPTp) with sulfadoxine-pyrimethamine (SP) as part of strategies to control malaria in most endemic countries (WHO, 2009). IPTp-SP involves the administration of a supervised curative treatment dose of SP at each scheduled antenatal care visit starting as early as possible in second trimester and at an interval not less than 4 weeks apart and up to the time of delivery (WHO, 2014). WHO recommended IPTp-SP as a strategy for prevention of malaria in pregnancy in 2001 but IPTp-SP was only adopted in 2005 as national policy in Nigeria (WHO., 2000, FMOH., 2005). The implementation of this strategy is being faced with challenges such as timing of SP administration (Onoka et al., 2012), knowledge and practices of the population (Onwujekwe et al., 2012, Diala et al., 2013) and rising levels of parasite resistance to SP in the general population (Happi et al., 2005, Mockenhaupt et al., 2008). Seasonal malaria chemoprevention (SMC) is another malaria control intervention, which uses SP. It is the administration of a complete treatment course of amodiaquine plus SP to children aged between 3 and 59 months at monthly intervals, beginning at the start of the transmission season to a maximum of four doses during the malaria transmission season (WHO, 2012). SMC is only recommended in areas with highly seasonal malaria transmission in the Sahel sub-region of sub-Saharan Africa, where *P. falciparum* is sensitive to both antimalarial medicines. SMC has been fully deployed in Katsina and Jigawa states of northern Nigeria.

Surveillance of SP resistance levels must be achieved by monitoring of molecular markers (WHO, 2004). SP resistance is linked with substitutions of amino acids in the enzymes dihydropteroate synthetase (*DHPS*) and dihydrofolate reductase (*DHFR*) in the folate biosynthetic pathway (Cowman et al., 1988, Triglia and Cowman, 1994, Brooks et al., 1994). Pyrimethamine targets the enzyme *DHFR* disrupting catalysis of the NADPH-dependent reduction of 7, 8-dihydrofolate to 5,6,7,8-tetrahydrofolate (Blakeley, 1984) while sulfadoxine blocks the folate biosynthetic pathway at the *DHPS* level by disrupting the coupling of 7,8,-dihydro-6-hydroxymethylpterin pyrophosphate with para-amino benzoic acid (pABA) to yield 7, 8- dihydropteroate (Walter, 1991).

Resistance to SP has evolved worldwide, and is caused by point mutations that accumulate at multiple sites in both the *dhfr* and *dhps* genes (Wang et al., 1997). In both genes, each successive mutation has been shown to incrementally increase the parasite's tolerance to the drug *in vitro* (Triglia et al., 1997, Triglia et al., 1998). An asparagine substitution at codon 108 of *dhfr* followed by substitution at codons 51 and 59 seem to be necessary for pyrimethamine resistance while an additional mutation at codon 164 (I164L) has been associated with high grade pyrimethamine resistance (Plowe et al., 1997). Mutations at codons 437 and 540 of *dhps* play the most significant role in sulfadoxine resistance among African parasites. In East and South Africa, mutations at the 437 and 540 codons are found together while in West and Central Africa the 437 is found on its own (Pearce et al., 2009). Laboratory studies show that the A437G and K540E substitutions in combination raise sulfadoxine tolerance of sensitive *DHPS* by 200 fold, compared to just 10 fold for the A437G substitution alone (Triglia et al., 1997). Hence East African parasites are predicted to withstand higher doses of SP than West African parasites. The efficacy of IPTp-SP is being further compromised in east Africa by the additional emergence of *dhps* mutation at codon 581 in northern Tanzania (Gesase et al., 2009) which has been shown to reduce the efficacy of IPTp-SP (Minja et al., 2013) termed super resistance (Naidoo and Roper, 2013). WHO recommended that prior to implementation of IPTp-SP in any region with moderate to high malaria transmission, the prevalence of K540E and A581G should be determined. IPTp-SP should be used in regions with a prevalence rate K540E less than 50% and A581G less than 10% (WHO, 2013a).

Hitherto the *dhps* K540E and A581G mutations have been rare in West and Central Africa and this is consistent with evidence of IPTp-SP efficacy during the same period (Falade et al., 2007, Aziken et al., 2011).

Reports of novel *dhps* mutations at codon 431(I431V) from UK imported malaria infections originating from Nigeria (Sutherland et al., 2009) and pregnant women from Cameroon (Chauvin et al., 2015) suggest this mutation is emerging. In Nigeria, there has been a dearth of molecular surveillance data (Naidoo and Roper, 2011, Drug resistance maps, http://www.drugresistancemaps.org/ipti/) which makes this difficult to substantiate. Crucially this needs to be addressed to underpin the continuing use of SP for IPTp and seasonal malaria chemoprevention (SMC).

In order to fill the gap in SP resistance surveillance data in Nigeria, we analysed retrospectively 1000 filter paper blood spots collected from malaria-infected pregnant women (with or without IPTp-SP intervention) and children with uncomplicated falciparum malaria from four geopolitical zones in Nigeria. We also evaluated the patterns of genetic changes in the parasite between 2003 and 2015. This study is the first of its kind providing patterns of SP resistance in different regions of Nigeria.

## 2 Materials and methods

**Fig. 1:**
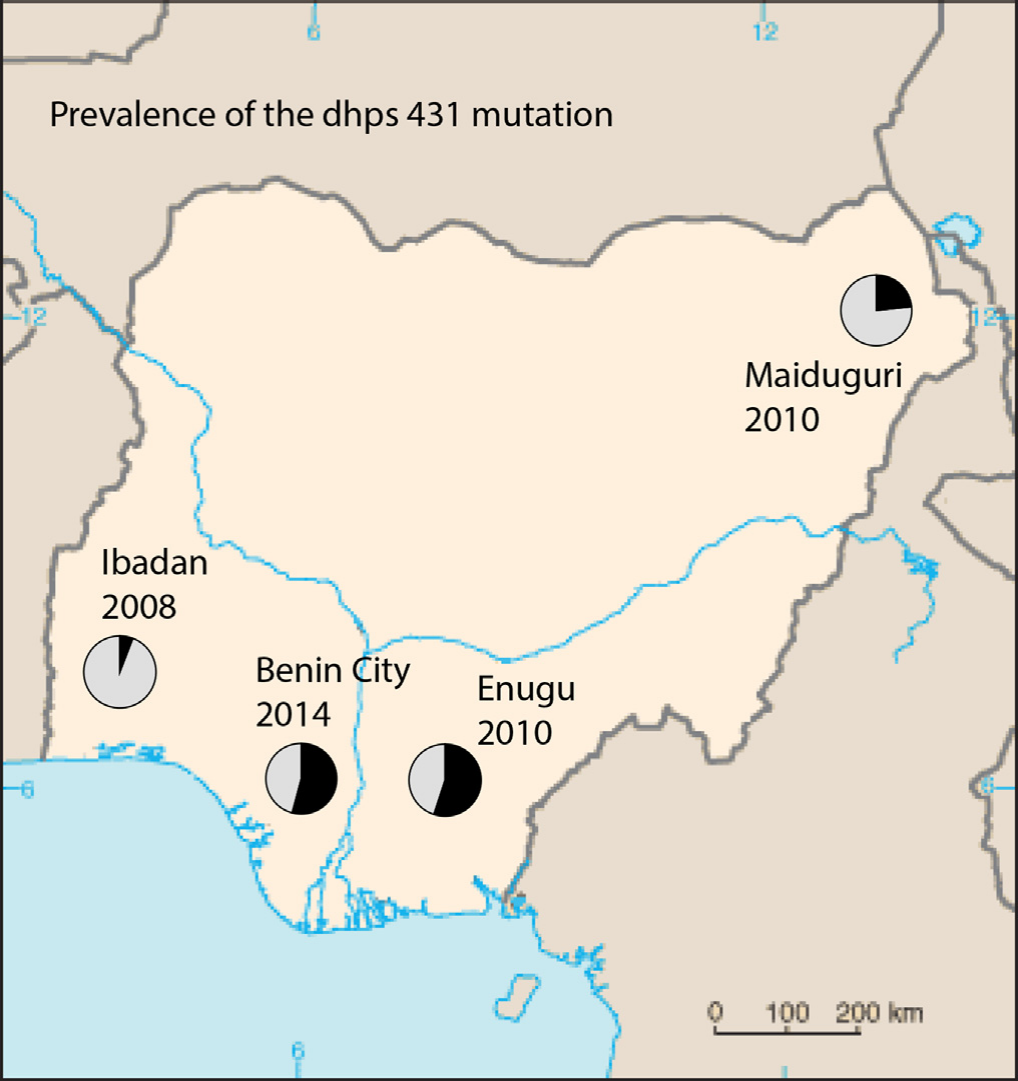
Prevalence of *dhps* 431 mutation haplotypes between 2003 and 2014 in Nigeria. A map of Nigeria showing the study locations and the prevalence of the various combinations of the *dhps*-431V haplotypes between 2003 and 2014/2015. The prevalence of *dhps*-431V haplotypes in Yaounde, Cameroon (2015) is also shown.

We identified molecular markers of SP resistance by nested PCR and direct sequencing in 1000 malaria positive blood spots collected from pregnant women and children attending hospitals across Southwest, Southeast, South-south and Northeast Nigeria (Fig. 1). Southern Nigeria comprises of the tropical rain forest with perennial malaria transmission occurring in rural and urban areas while the northern part is mostly characterized as arid savannah with less annual rainfall and more seasonal transmission (Ekanem et al., 1990). In the past, chloroquine and sulfadoxine-pyrimethamine were used but later abandoned in 2005 due to increased threat of drug resistance. The antimalarial drug regimens for all parts of Nigeria is currently artemether-lumefantrine and amodiaquine-artesunate. Filter paper blood spots and placental impressions were collected from pregnant women attending St Mary's Catholic Hospital Eleta Ibadan between May 2003 and October 2004 (Falade et al., 2007), Damboa General hospital between 2010 and 2012 (Damboa LGA, Borno State), Polyclinic (an extension of Park Lane hospital) and Balm of Gilead Specialist hospital between 2010 and 2012 (both in Enugu, Enugu State). Filter paper blood spots were also collected from children with uncomplicated malaria presenting at General Outpatient Department of the University College Hospital (UCH), Ibadan, and the Primary Health Care Center (PHC), Idi-Ayunre, Oluyole Local Government Area (both in Oyo State) between August 2007 and May 2008 (Falade et al., 2014); University of Benin Teaching Hospital (UBTH) and Sickle-cell centre (both in Edo State) between September 2014 and April, 2015 (details of this study is being prepared for a separate publication). Oyo state is located in Southwest, Borno state in Northeast, Enugu state in Southeast and Edo state in South-south geopolitical zones of Nigeria. Informed consent was obtained from all subjects before enrolment. All filter paper blood samples were shipped to London School of Hygiene and Tropical Medicine for molecular analysis. Ethical approval for the 2003 study was obtained from the University of Ibadan/University College Hospital Institutional Review Committee and the Boston University Institutional Review Board. The 2007 study was approved by the University of Ibadan/UCH Institutional Review Committee and Oyo State ministry of health ethical review committee. The 2010 study was approved by the Health Research Ethics Committee of the University of Nigeria Teaching Hospital, Ituku-Ozalla, Enugu while the 2015 study was approved by the Ethics and Research community, University of Benin Teaching hospital and Hospital Management Board.

### 2.1 DNA preparation, PCR diagnosis, PCR genotyping and sequencing

#### 2.1.1 DNA preparation

DNA extraction from blood spots was carried out in 96-well plate format using the Chelex extraction method as described elsewhere (Plowe et al., 1995).

#### 2.1.2 Description of study samples

DNA was extracted from a total of **1000** filter paper blood spots. In Ibadan, samples from two different time points (2003 and 2007) were used. In 2003, fifty (50) matched maternal and placental blood spots (100 individual samples) from pregnant women were evaluated. In 2007–2008, two hundred (**200**) filter paper blood spots obtained at enrolment from children with uncomplicated falciparum malaria were evaluated. In 2010–2012, samples obtained from an in vivo efficacy study of IPTp-SP in Maiduguri and Enugu (Component A) were evaluated. A further subset from a cross-sectional study of pregnant women in Enugu (Component B) were also evaluated. In Maiduguri, a total of one hundred and forty-two (**142**) filter paper blood spots were evaluated. These consisted of day0, 7, 14, 21, 28, 42, maternal blood and placental samples from each sample ID. Likewise in Enugu (Component A), a total of two hundred and thirty-three (**233**) samples were evaluated from day0, 7, 14, 21, 28, 42, maternal and placental blood spots. For Enugu (Component B), two hundred and twenty-five (**225**) samples were evaluated. In 2014–2015, one hundred (**100**) samples from a cross-sectional survey amongst sickle cell and normal children were evaluated. Components A and B were the followed up (FU) and non-followed up (Non-FU) pregnant women, respectively.

#### 2.1.3 PCR diagnosis, PCR genotyping and sequencing

One thousand samples were screened for infection with *P. falciparum* using nested PCR (Snounou et al., 1993). Point mutations at codons 16, 50, 51, 59, 108, 140 and 164 of the *dhfr* gene and codons 431, 436, 437, 540, 581 and 613 of the *dhps* gene were evaluated by nested PCR amplification as earlier described (Pearce et al., 2003). 594 bp and 711 bp products of *dhfr* and *dhps* genes respectively were sized against 100 bp molecular weight marker on 1.2% agarose gel stained with ethidium bromide. PCR products were enzymatically purified using Exonuclease I-Fast Alkaline Phosphatase according to the manufacturer's instructions followed by direct sequencing of products. Sequences were analysed using Lasergene analysis software (DNAStar, Madison, WI).

### 2.2 *P. falciparum* DHPS modelling

#### 2.2.1 Preparing the intensive homology model of PfDHPS

A modified Phyre2 model (Kelley et al., 2015) was developed for the *P. falciparum* IT (wild type enzyme) using the intensive option. The sequence ran from just before the start of the *DHPS* sequence at residue Ile 366 to the C-terminus at Val 706. 11 May 13.33 2016 (ref:0347ffe1a64d64b1). The program chose 6 template crystal structures for the final modelling process as follows:

1 AD1 (a) *Staphylococcus aureus*
1 AJZ (a) *Escherichia coli*
3 TR9 (a) *Coxiella burnetti*
1 EYE (a) *Mycobacterium tuberculosis*
2 BMB (a) *Saccharomyces cerevisiae*
1 TX2 (a) *Bacillus anthracis*

Eighty-nine percent (89%) of residues were modelled at high (>90%) confidence but confidence in the second *P. falciparum* insert towards the end of the sequence 624–666 was low. The QMEAN test (Benkert et al., 2009) was used to determine the reliability of the modelled protein. The QMEAN value was 0.567 with a Z-score of −2.43 (“estimated reliability between 0 and 1”).

#### 2.2.2 Introduction of single mutations using the site directed mutator “DUET” (Pires et al., 2014)

DUET can be used on homology-modelled or crystal structures to check the impact of individual drug-resistance mutations, in addition to allowing examination of structures in the modified *.pdb file in pdb viewers.

The Program works by measurement of Gibbs energy change (delta delta Gibbs, δδG) (in kilocalories/mol) involved in folding and unfolding a wild type or a mutated structure, taking the overall energy involved for the wild type as zero, and comparing the increase or decrease of energy seen in the same process for the mutant. This gives a positive (stabilizing) value or a negative (destabilizing) value for the new residue. Mutations having very positive or very negative δδG values are likely to render a protein less fit than where δδG is moderate, so the values expected for most common non-damaging mutations are of the order of + or - 1.0. It follows that under natural conditions the advantage gained by the organism, after residue change, will depend very much on balancing the intrinsic loss of fitness with the intensity of drug-pressure.

## 3 Results

Point mutations in *dhps* and *dhfr* genes were evaluated in 1000 malaria-positive (PCR-confirmed) filter paper blood spots obtained from pregnant women and children in the southern and northern parts of Nigeria between 2003 and 2015. In Ibadan (2003), of the fifty (50) matched maternal and placental blood spots evaluated, thirty-eight (38) were successfully amplified and sequenced. In 2007–2008, of the two hundred (200) filter paper blood spots evaluated, 198 were successfully amplified and sequenced for *dhps* while 191 were successfully sequenced for *dhfr* mutations. In Maiduguri (2010–2012), of the one hundred and forty-two (142) filter paper blood spots evaluated, 53 and 48 samples were used for *dhps* and *dhfr* analyses respectively. A sample ID with similar haplotypes on day0, 7, 14, 21, 28, 42, maternal or placental were counted as one haplotype. Likewise, in Enugu (Component A), of the 233 samples evaluated from day0, 7, 14, 21, 28, 42, maternal and placental blood spots, 145 samples were left for *dhps* and 139 for *dhfr* analyses after filtering off the duplicates by sample IDs. For Enugu (Component B), of the two hundred and twenty five (225) evaluated only 60 (maternal or placental – duplicate haplotypes were dropped) blood spots were successfully sequenced for *dhps* and 51(maternal or placental) blood spots for *dhfr*. In 2014–2015, of the one hundred samples evaluated, ninety-five (95) and seventy-nine (79) were successfully sequenced for *dhps* and *dhfr* mutations respectively. In all, a total of 589 and 546 analysable data were obtained for *dhps* and *dhfr* mutations respectively. It is noteworthy that the nested Snonou protocol for malaria diagnosis is highly sensitive compared to the nested PCR protocols for *dhps* and *dhfr* gene amplification and as such fewer successful sequence data were obtained for the pregnant women.

### 3.1 Prevalence of dhps haplotypes

Table 1 shows the prevalence of *dhps* haplotypes from the various geographical regions between 2003 and 2015. Of the 38 successfully sequenced isolates from Ibadan in 2003, seventy-eight percent (78%) were of the single mutant *dhps* haplotype ISGKAA, 10% were of the IAGKAA, 5% IAGKAS and another 5% comprised of mixed haplotypes. No *dhps* 431V haplotype was observed in 2003. In Ibadan (2007), of the 198 successfully sequenced isolates, sixty-one percent (61%) harboured the single mutant ISGKAA, 4% IAGKAA, 5.1% IAGKAS, 16.2% mixed haplotypes, 5.6% IAAKAA, 0.5% with double mutant *dhps* ISGEAA and 0.5% of other minor haplotypes. There was an observed increase of 5% 431V haplotype – 3% VAGKAA, 1% VAGKAS and 1% VAGKGS. In Maiduguri (2010), of the 53 isolates analysed, 20.8% harboured the single mutant ISGKAA, 3.8% IAGKAA, 3.8% mixed haplotypes, 39.6% IAAKAA, 3.8% IFAKAS, 1.9% ISAKAA (wild-type), 1.9% IAAKGS and 1.9% ICAKAA. A prevalence of 22.6% was observed for the 431V haplotypes – 11.3% VAGKAA and 11.3% VAGKGS. In Enugu (2010- Component A), of the 145 analysable samples, 25.5% harboured the ISGKAA, 6.2% IAGKAA, 9.6% mixed haplotypes, 1.4% IAAKAA, 0.7% IAAKGA and 2.1% IFAKAS. A total prevalence of 54.5% was observed for the 431V haplotypes - 6.9% VAGKAA, 1.4% VAGKAS and 46.2% VAGKGS. In the component B study – Enugu 2010, of the 60 successfully analysed samples, 40% harboured the ISGKAA, 1.7% IAGKAA, 3.3% IAGKAS, 6.7% mixed haplotypes, 6.7% IAAKAA and 1.7% ISAKAA. The 431V haplotype was observed in 40% of the isolates and these were all VAGKGS. In Benin City (2015), of the 95 successfully sequenced isolates, 30.5% harboured ISGKAA, 4.2% IAGKAA, 2.1% IAGKAS, 1.1% mixed haplotypes, 3.2% IAAKAA and 1.1% of other haplotypes. The 431V haplotype occurred in more diverse forms with a total prevalence of 53.8%–2.1% VAGKAA, 3.2% VAGKAS, 7.4% VAGKGA, 1.1% VSGKAA, 1.1% VSGKGA, 2.1% VSGKGS and 36.8% VAGKGS.

### 3.2 Prevalence of dhfr haplotypes

The prevalence of *dhfr* haplotypes in the various regions between 2003 and 2015 are shown in Table 2 with no significant change observed across the sites. In Ibadan (2003), all of the 38 successfully sequenced isolates harboured the triple mutant *dhfr* ACIRNVI. In 2007, 96.9% of the 191 isolates harboured the triple mutant ACIRNVI, 0.5% ACICNVI, 0.5% ACNCSVI (wild type), 1.6% ACNRNVI and 0.5% mixed haplotypes. In Maiduguri (2010), of the 48 isolates, 81.3% harboured ACIRNVI, 4.2% ACNCSVI, 10.4% ACNRNVI and 4.2% mixed haplotypes. In Enugu (Component A- 2010), of the 139 isolates, 94.2% harboured ACIRNVI, 0.7% ACICNVI, 0.7% ACNRNVI, 2.9% mixed haplotypes and 1.4% ACNCNVI. In Enugu (Component B), of the 51 isolates, 90.2% harboured ACIRNVI, 2% ACICNVI, 3.9% ACNCSVI and 3.9% mixed haplotypes. In Benin City, 98.7% of the 79 isolates harboured ACIRNVI while 1.3% had ACICNVI. The triple mutant *dhfr* is predominantly high across all years with 100 percent frequency observed in 2003. No mutations were observed at codons 16, 50, 140 and 164 of the *dhfr* gene.

### 3.3 Prevalence of dhps alleles

Table 3 summarizes the prevalence of the individual *dhps* alleles (codons 431, 436, 437, 540, 581 and 613). The allelic frequencies for studies in pregnant women were split into maternal (M) and placental (P) samples. The prevalence of placental parasitaemia was very low in 2003 and 2010 except for Component B (Enugu). This is expected since Component B was a non-intervention arm with most women not actively taking IPTp-SP and as a result the level of protection from placental parasitaemia was low. Fig. 1 shows a descriptive map of Nigeria with the emergence of *dhps*-VAGKGS haplotype and a complete absence of 431V-haplotype in 2003 (Ibadan). It also highlights the prevalence of 431V-haplotype in Yaounde, Cameroon previously described by Chauvin et al. (2015).

### 3.4 A homology model of *P. falciparum* DHPS

**Fig. 2:**
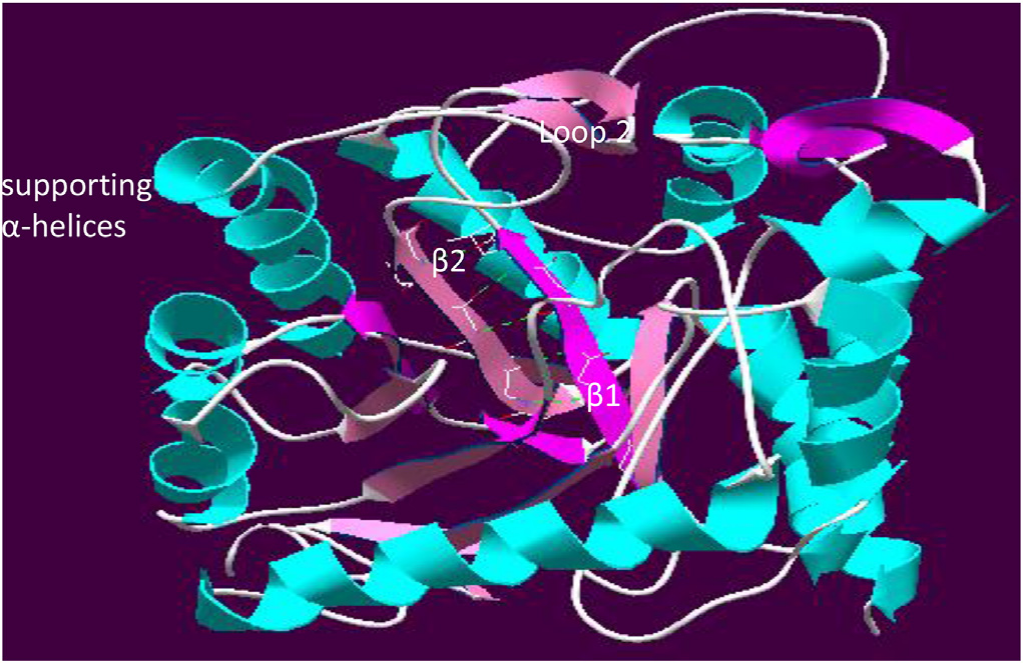
**Full model *Pf*DHPS showing H-bonded parallel β-strands 1 and 2**. At the C-terminus of β-2 is loop 2 containing substrate-binding mutable residues Ser436 and Ala437.

Fig. 2 shows the full model *Pf*DHPS showing H-bonded parallel β-strands 1 and 2. At the C-terminus of β-2 is loop 2 containing substrate-binding mutable residues Ser436 and Ala437. The homology model of PfDHPS suggests the probable effect of the I431 to 431V change. Supplementary Fig. 1 describes β-1 and β-2 strands of *Pf*DHPS in the wild type I431 and mutant type 431V respectively. In our homology model for the wild-type *Pf*DHPS component, the side chain of Ile 431 in β-2 shows hydrophobic interaction (2.763 Å) with that of Leu 395 in β-1. The Ile 431 Val change is expected to prevent this inter-strand stabilizing hydrophobic interaction since the closest side-chain approach is 4.157 Å. This may render the substrate-binding residues in loop **2** marginally less stable, enhancing resistance to drug inhibition.

**Fig. 3:**
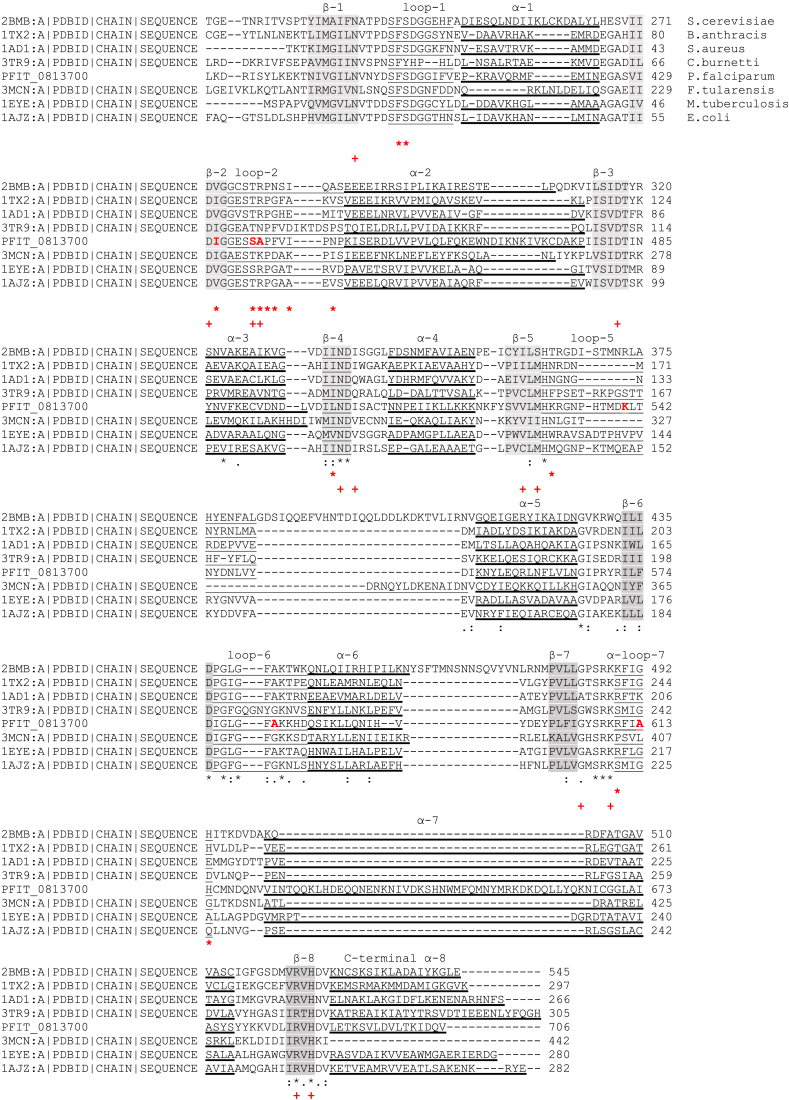
Clustal-0 alignment of DHPS sequences to locate structural features. This is largely from Pemble et al. (2010) but sequences used, apart from *P. falciparum*, are from FASTA Texts published with the crystal structures.

Fig. 3 shows the clustal alignment of DHPS sequences to locate structural features. This alignment is largely from Pemble et al., (2010) but sequences used, apart from *P. falciparum*, are from FASTA texts published with the crystal structures. The reactive enzymatic parts of the DHPS (dihydropteroate synthase) structure are enclosed in a supporting TIM Barrel (**T**riose phosphate **I**so**M**erase Barrel structure) which is a conserved protein fold consisting of eight α-helices () in the alignment. Inside are eight β-strands (shaded) (arranged in 4, backbone H-bonded, parallel pairs) that alternate with the α-helices along the peptide backbone. The drug-binding DHPS residues associated with sulphonamide resistance (*) are seen almost exclusively in flexible loops (underlined), which are located between the relatively-rigid β-strands and the α-helices. Substrate-binding sites **(+)** are found in the loops and in β-strands.

Although the genus Plasmodium is atypical in having a bifunctional HPPK/DHPS where, as seen in the yeasts Francisella (Pemble et al., 2010) and Saccharomyces (Lawrence et al., 2005) DHPS is fused to the preceding enzyme in the folate synthetic pathway, 6-hydroxymethyl-7,8-dihydropterin pyrophosphokinase (HPPK) (de Beer et al., 2006). The 3D structure in both HPPK-fused and separate DHPS is quite typical.

In the homology model of *Pf*DHPS the β-strands fit readily into the typical picture but where earlier resistance-associated residue changes have only been seen in flexible loops, Ile431Val is seen in a beta-strand, β-2, preceded by the completely conserved Asp 430 residue, a substrate-binding site and followed by the completely-conserved Gly 432. β-1 and β-2 are structurally adjacent parallel β-strands, H-bonded together by backbone atoms into a rigid β-sheet. In β-1 of our homology model for the wild-type *Pf*DHPS component, the bulky side chain of Ile 431 in β-2 hydrophobically interacts with the side chain of Leu 395 in β-1 (Supplementary Fig. 1). This strengthens the link between C-terminal ends of these β-strands, participating in stabilizing the position of the mutable substrate-binding residues Ser 436 and Ala 437 in flexible Loop-2.

In the *Pf*DHPS sequence, the residues subject to mutation are highlighted in red (Fig. 3). Additionally, in β-1, completely conserved Asn 396, follows hydrophobically interacting Leu 395 directly, and this interaction is lost in I431V. The mutation may very likely affect N 396 in its role as a substrate-binding site, as well as making the positions of drug-interacting and substrate-binding residues in the attached loop less fixed. There is only one resistance-associated residue change in a β-strand in the DHPS alignment of Pemble et al., (among 12 noted overall), which is in β-4, the equivalent WT residue in β-4 in the PfDHPS structure being Leu 501, next to completely conserved Asn 502 and Asp 503, the first of which is a substrate binding site.

Table 4 shows the Gibbs energy change (delta delta Gibbs, δδG) (in kilocalories/mol) involved in folding and unfolding a wild type or a mutated structure. The values reflect the degree of stabilizing or destabilizing effect of the changes. The I431V has a δδG value of −1.622 which is similar to that of A581G (−1.64) and A613S (−1.626) with destabilizing effects.

## 4 Discussion

In this study of SP resistance markers in Nigeria during 2003–2015, we observed an emergence of the *dhps* I431V mutation in Ibadan during 2003–2008. The I431V mutation was first described in a study by Sutherland and others (2009) in isolates from malaria patients returning to the UK from Nigeria. It has since been reported in neighbouring Cameroon (Chauvin et al., 2015) at a prevalence of 9.8% but not among the numerous *dhps* sequencing studies conducted on parasite populations in the rest of Africa (Naidoo and Roper, 2013). Published reports of the mutation are currently confined to Nigeria and Cameroon although one appears in a Ghanaian isolate among publicly available whole genome sequences (Plasmoview, http://pathogenseq.lshtm.ac.uk/plasmoview). Early sequencing surveillance studies of *dhps* in Cameroon (Tahar and Basco, 2007) did not detect the 431V among 355 samples collected in multiple sites (Yaounde, Djoum, Manjo, Bertoua and Garoua) during 1999–2003 which suggests that the appearance of this mutation is comparatively recent. Reports of the prevalence of the 431V are mapped in Fig. 1.

The I431V was seen in combination with various other *dhps* mutations but interestingly was most abundant in combination with 437G, 581G and 613S in the VAGKGS haplotype. The haplotype prevalences summarised in Fig. 1 show the VAGKGS haplotype was more prevalent in Enugu (in 2010) than Maiduguri (in 2010) and was found at high prevalence in Benin City (2014). The increase in mutant *dhps*-VAGKGS over 12 years seems to indicate that it confers a selective advantage in the presence of SP drug pressure and is displacing more sensitive haplotypes. As well as IPTp, SP is still used for the treatment of malaria and is readily available in the Nigerian market (Ugwu et al., 2013) so ongoing SP drug pressure is strong. Importantly, the *dhps*-VAGKGS and VAGKAA were also widely dispersed being found in Maiduguri which is far from the other sites and this indicates that the haplotype may be spreading throughout Nigeria.

The *dhps*-540E mutation was observed at low prevalence (0.5%) in Ibadan in 2007/2008, it was otherwise absent. Contrastingly, we observed a significant increase in the prevalence of 581G and 613S over the same time period. Prevalence increased from 0% in 2003 to 52.6% in 2014 for *dhps*-581G and 8%–51.6% in 2014 for *dhps*-613S. A significant part of this expansion could be accounted for by the increase in the VAGKGS haplotype.

The triple mutant *dhfr*-IRN was almost fixed across the years with prevalence above 90%. Although the quintuple *dhfr*/*dhps* mutation was almost absent in this study, the combination of IRN with alternative *dhps* resistance haplotypes to the east African GEA and GEG (437 + 540 + 581 mutant haplotypes) may be conferring increased SP tolerance levels.

### 4.1 What is the significance of dhps 431V mutations for efficacy of SP?

Models of the 3-dimensional structure of the DHPS molecule can be used to explore how the I431V substitution might affect resistance. The mutation involves a change from isoleucine to valine in a highly conserved region of the DHPS molecule, close to mutable residues S436 and A437. The preceding conserved residue D430 is recognised as a substrate-binding site. Valine is less hydrophobic than isoleucine and its side-chain is smaller than that of isoleucine.

Although we do not yet have conclusive evidence that there is an effect of the β-2 I431V change on drug response for *Pf*DHPS, the comparison with the effective mutation signalled by Pemble et al. in β-4 is suggestive. In crystal 3MCNa, of *F. tularensis* HPPK/DHPS, the side chain of Ile 276 in β-4 hydrophobically interacts (3.59 Å) with that of Ile 291 in α-4 (this interaction is lost on mutation to Val) and with side chain of Met262 (2.97 Å) in α-3, which is retained after the Ile 276 Val mutation. Comparing the calculated DUET δδG values of mutations (Table 4) we find that in β-2 of structure *Pf*DHPS and β-4 of crystal 3MCNa the mutations I431V and I276V show essentially the same destabilizing δδG of −1.62 kcal/mol. This is another hint that a similar effect is perhaps being registered in both cases.

The arrangement and association of β-strands in the *Pf*DHPS structure is of interest, because there are 2 large parasite-specific inserts in the overall sequence (in α-2 and α-7) which might be expected to have a disruptive effect. The parallel backbone H-bonding of β-1 to β-2, β-3 to β-4, β-5 to β-6 are clearly defined, but β-7 to β-8, where β-8 follows α-7, has only 3 H-bonds. de Beer et al. concluded that the presence of the Plasmodium-specific inserts was probably functional and they largely avoided deleting them, although it was necessary for some of their molecular dynamic procedures. The illuminating results obtained by de Beer et al. have undoubtedly been invaluable in extending our understanding of the *Pf*DHPS active site and the effects of mutations. In our short study the direct effects of mutations have so far only been glanced at. At least allowing the inserts to remain has not prevented our homology model from achieving the Q-mean criteria for an acceptable structure.

The efficacy of IPTp-SP remains high in Nigeria irrespective of the high prevalence of quadruple mutant *dhfr*/*dhps* mutation (Falade et al., 2007, Aziken et al., 2011). We think/hypothesize that the *dhps*-VAGKGS is highly resistant and may be associated with low birthweight, high placental parasitaemia at birth and less likely to clear infections compared to the wild type *dhps*-ISAKAA. Another plausible explanation is that VAGKGS has arisen by chance and provides an improvement in the fitness of parasites carrying the 437, 581 and/or 613 mutations, but does not of itself change susceptibility to sulfadoxine. Thirdly, VAGKGS has been selected by other drugs, such as the antibacterial sulpha drug sulphamethoxazole, but does not directly impact on susceptibility to SP. Further studies need to be carried out to test the validity and importance of these hypotheses as there is lack of information on the phenotype of parasite carrying this haplotype.

### 4.2 Limitations of the study

A major limitation of this study is the difference in sampling sites. Although the transmission patterns in Enugu, Ibadan and Benin City are similar, there is lower prevalence and strict seasonality of malaria in Maiduguri (northeast Nigeria). Another limitation of the study is that we did not take into account HIV-infected women and children who may already be on co-trimoxazole (septrin). Co-trimoxazole is given to HIV-infected patients and HIV-exposed but not infected children in order to prevent opportunistic infections (WHO., 2013b, Bwakura-Dangarembizi et al., 2014). It is known to have significant antimalarial activity (Omar et al., 2001) but is contraindicated in patients using SP. Studies have shown that although co-trimoxazole like SP, selectively targets dihydrofolate reductase (*DHFR*) and dihydropteroate synthetase (*DHPS*), it selects *dhfr*-IRS haplotype and *dhps*-A437 and A581 alleles (Jelinek et al., 1999).

### 4.3 Conclusion

Our homology model of *P. falciparum* DHPS suggests a probable effect of the Isoleucine 431 to Valine change. Although, this does not specifically indicate that the 431V is the West African way of bypassing the importance of the 540E, it is highly suggestive that the I431V would prevent the inter-strand stabilizing hydrophobic interaction between the β strands. Consequently, the change to 431 Valine will likely disrupt sulfadoxine binding to the active site and it is probable that this effect is compounded by changes in amino acids at codons 436, 437, 581 and 613. Our data so far serves as baseline surveillance of molecular determinants of SP resistance relevant to the use of SMC in Maiduguri, Borno state and other qualifying states. Based on our findings, it has become crucial to evaluate the impact of *dhps*-VAGKGS and other combinations of 431V in SMC and IPTp since this emerging mutation is on the increase. More tailored studies to address this question are currently underway.

## Figures and Tables

**Table 1:** Prevalence of *dhps* haplotypes in Nigeria (2003–2015).

*dhps* haplotype	Ibadan 2003 (%)	Ibadan 2007/8 (%)	Maiduguri 2010 (%)	Enugu FU 2010 (%)	Enugu non-FU 2010 (%)	Benin city 2014/15 (%)
ISGKAA	**30** (78.9)	**121** (61.1)	**11** (20.8)	**37** (25.5)	**24** (40)	**29** (30.5)
IAGKAA	**4** (10.5)	**8** (4)	**2** (3.8)	**9** (6.2)	**1** (1.7)	4 (4.2)
IAGKAS	**2** (5.3)	**10** (5.1)			**2** (3.3)	**2** (2.1)
MIXED	**2** (5.3)	**32** (16.2)	**2** (3.8)	**14** (9.6)	**4** (6.7)	**1** (1.1)
IAAKAA		**11** (5.6)	**21** (39.6)	**2** (1.4)	**4** (6.7)	**3** (3.2)
IAAKGA		**1** (0.5)		**1** (0.7)		
IFAKAS		**1** (0.5)	**2** (3.8)	**3** (2.1)		
ISAKAA		**1** (0.5)	**1**(1.9)		**1** (1.7)	
ISGEAA		**1** (0.5)				
ISGKAS		**1** (0.5)				**1** (1.1)
IYAKAS		**1** (0.5)				
IAAKGS			**1** (1.9)			
ICAKAA			**1** (1.9)			
IAGKGA						**1** (1.1)
IAAKGS						
IAGKGS						**1** (1.1)
ISGKGA						**1** (1.1)
ISGKGS						**1** (1.1)
VAGKAA		**6** (3)	**6** (11.3)	**10** (6.9)		**2** (2.1)
VAGKAS		**2** (1)		**2** (1.4)		**3** (3.2)
**VAGKGS**		**2 (1)**	**6 (11.3)**	**67 (46.2)**	**24 (40)**	**35 (36.8)**
VAGKGA						**7** (7.4)
VSGKAA						**1** (1.1)
VSGKGA						**1** (1.1)
VSGKGS						**2** (2.1)
VAAKAA						
**TOTAL**	**38**	**198**	**53**	**145**	**60**	**95**

FU – followed up; non−FU – non follow up.

Bold means actual figures while normal text indicates percentages.

**Table 2:** Prevalence of *dhfr* haplotypes in Nigeria (2003–2015).

*dhfr* haplotype	Ibadan 2003 (%)	Ibadan 2007/8 (%)	Maiduguri 2010 (%)	Enugu FU 2010 (%)	Enugu non-FU 2010 (%)	Benin city 2014/15 (%)
ACIRNVI	**38** (100)	**185** (96.9)	**39** (81.3)	**131** (94.2)	**46** (90.2)	**78** (98.7)
ACICNVI		**1** (0.5)		**1** (0.7)	**1** (2)	**1** (1.3)
ACNCSVI		**1** (0.5)	**2** (4.2)		**2** (3.9)	
ACNRNVI		**3** (1.6)	**5** (10.4)	**1** (0.7)		
MIXED		**1** (0.5)	**2** (4.2)	**4** (2.9)	**2** (3.9)	
ACNCNVI				**2** (1.4)		
**TOTAL**	**38**	**191**	**48**	**139**	**51**	**79**

**Table 3:** Summary of *dhps* alleles in 4 different regions of Nigeria (2003–2015).

*dhps* allele	Ibadan 2003 n = 38		Ibadan 2007/8 n = 198	Maiduguri 2010 n = 53		Enugu FU 2010 n = 145		Enugu non-FU 2010 n = 60		Benin city 2014/15 n = 95
	M	P		M	P	M	P	M	P	
I431	36	2	188	38	3	60	5	20	15	48
V431			13	11	2	89	2	18	8	52
S436	31	1	153	12	1	43	5	16	11	39
A436	7	1	70	34	4	108	3	19	16	58
F436			1	2		7	2			
C436				1						
Y436			1							
A437			24	26	2	11	2	6	1	3
G437	36	2	183	23	3	133	6	32	23	92
K540	36	2	197	48	5	139	6	38	22	95
E540			2							
A581	36	2	195	43	4	73	5	20	16	48
G581			5	6	1	74	2	18	8	50
A613	35	1	179	41	4	66	3	23	11	50
S613	2	1	24	8	1	82	2	17	10	49

M – Mother; P – Placenta.

**Table 4:** DUET δδG effects of mutations in *DHPS*.

Mutation	DUET δδG	Chain	Organism	Comment on effect	Site
I431V	−1.622	A	*P. falciparum*	Destabilizing	β-2 strand
S436A	−0.053	A	”	Destabilizing (very low)	Loop
S436F	−0.91	A	”	Destabilizing	Loop
A437G	−0.444	A	”	Destabilizing	Loop
K540E	0.204	A	”	Stabilizing (low)	3(10) helix
A581G	−1.64	A	”	Destabilizing	bend
A613S	−1.626	A	”	Destabilizing	α-helix
A613T	−1.491	A	”	Destabilizing	α-helix
I276V	−1.62	A	*Francisella tularensis*	Destabilizing	β-4 strand

Mutations having very positive or very negative δδG values are likely to render a protein less fit than where δδG is moderate, so the absolute values expected for most.

Common non-damaging mutations are of the order of + or − 1.0.

## Appendix A Supplementary data

Supplementary Fig. 1 **Models of *P. falciparum* DHPS wild type I431 in** β-2 **and mutant I431V**. The wild type Ile side-**c**hain shows hydrophobic interaction with side chain of Leu 395 in β-1 which is lost when Ile is replaced by Val. The proximity of the mutable residues 436 and 437 in flexible loop 2 to this destabilization of the relationship between the C-terminal regions of β-1 and β-2 is clearly seen. Supplementary Fig. 1

Supplementary Fig. 2 WTPfDHPSA.pdb file. Supplementary Fig. 2
