# Supplementary material for: Molecular determinants of sulfadoxine-pyrimethamine resistance in Plasmodium falciparum in Nigeria and the regional emergence of dhps 431V
Source: Int J Parasitol Drugs Drug Resist. 2016 Sep 29;6(3):220–9. doi: 10.1016/j.ijpddr.2016.08.004 (PMC5094156; doi:10.1016/j.ijpddr.2016.08.004)
Supplement: Supplementary Fig. 1 — Models of P. falciparum DHPS wild type I431 in β-2 and mutant I431V. The wild type Ile side-chain shows hydrophobic interaction with side chain of Leu 395 in β-1 which is lost when Ile is replaced by Val. The proximity of the mutable residues 436 and 437 in flexible loop 2 to this destabilization of the relationship between the C-terminal regions of β-1 and β-2 is clearly seen. [file mmc1.pptx]

## Slide 1
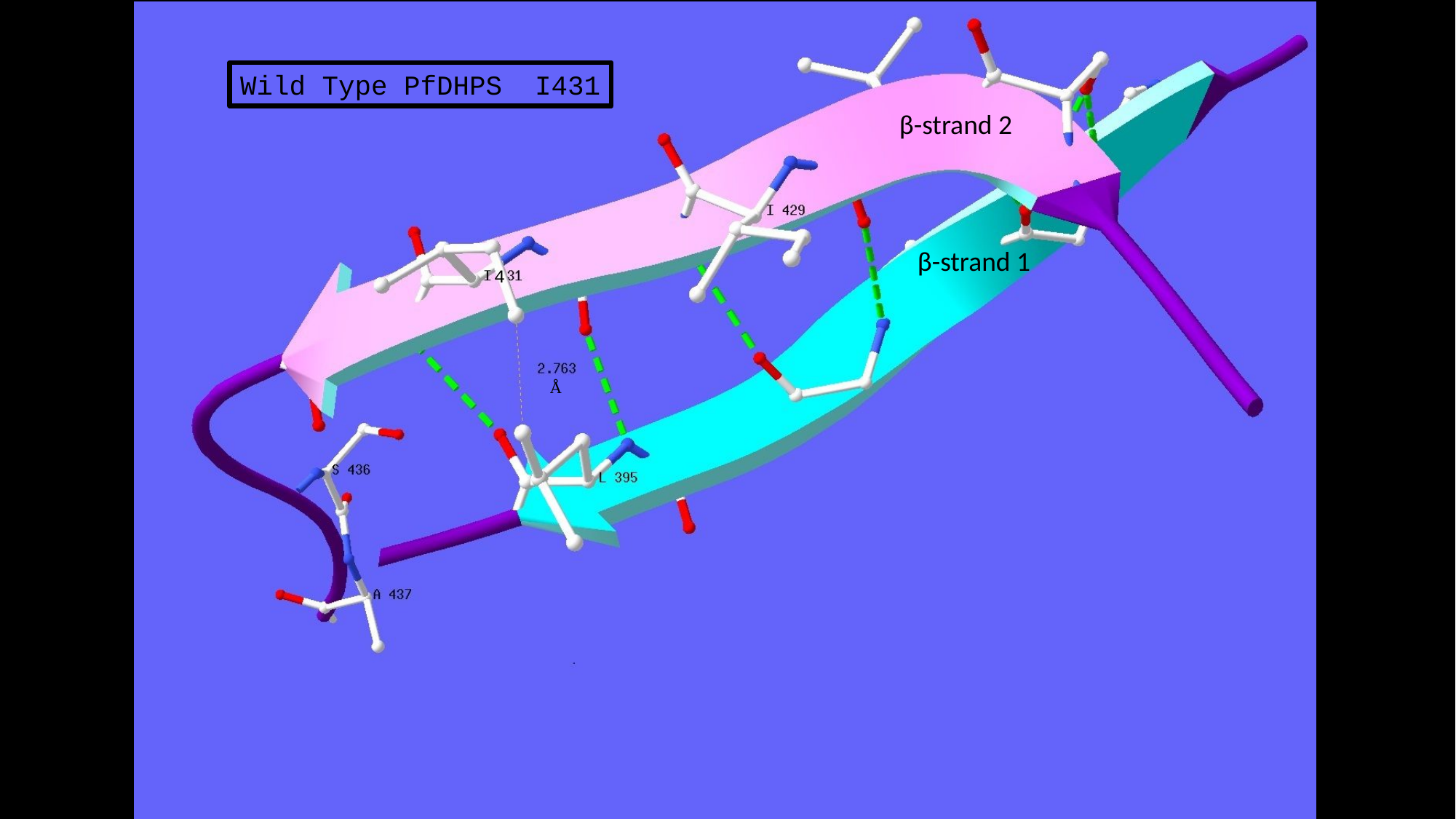

Wild Type PfDHPS I431
β-strand 2
β-strand 1
4
Å

## Slide 2
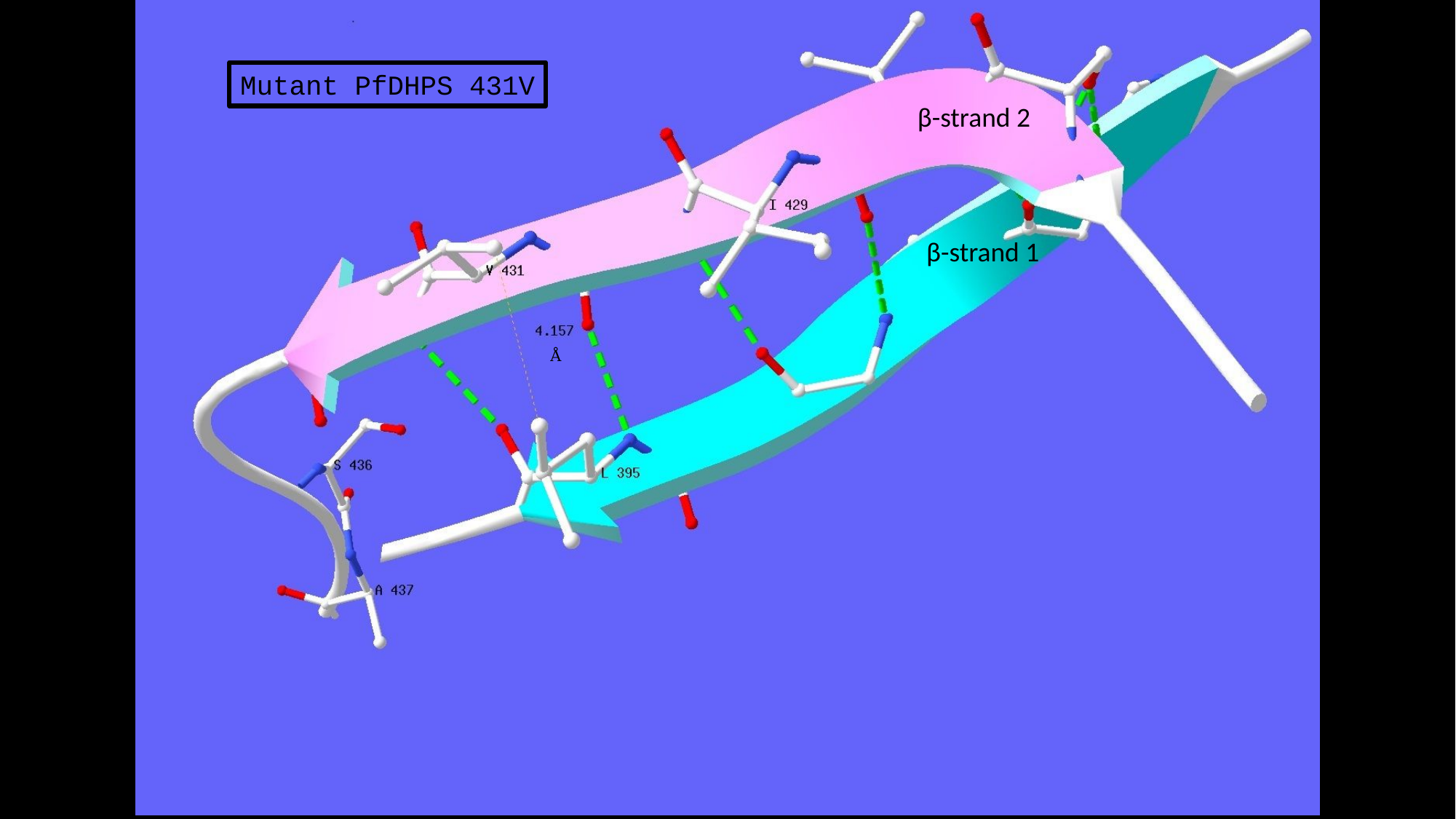

Mutant PfDHPS 431V
β-strand 2
#
β-strand 1
Å

## Slide 3
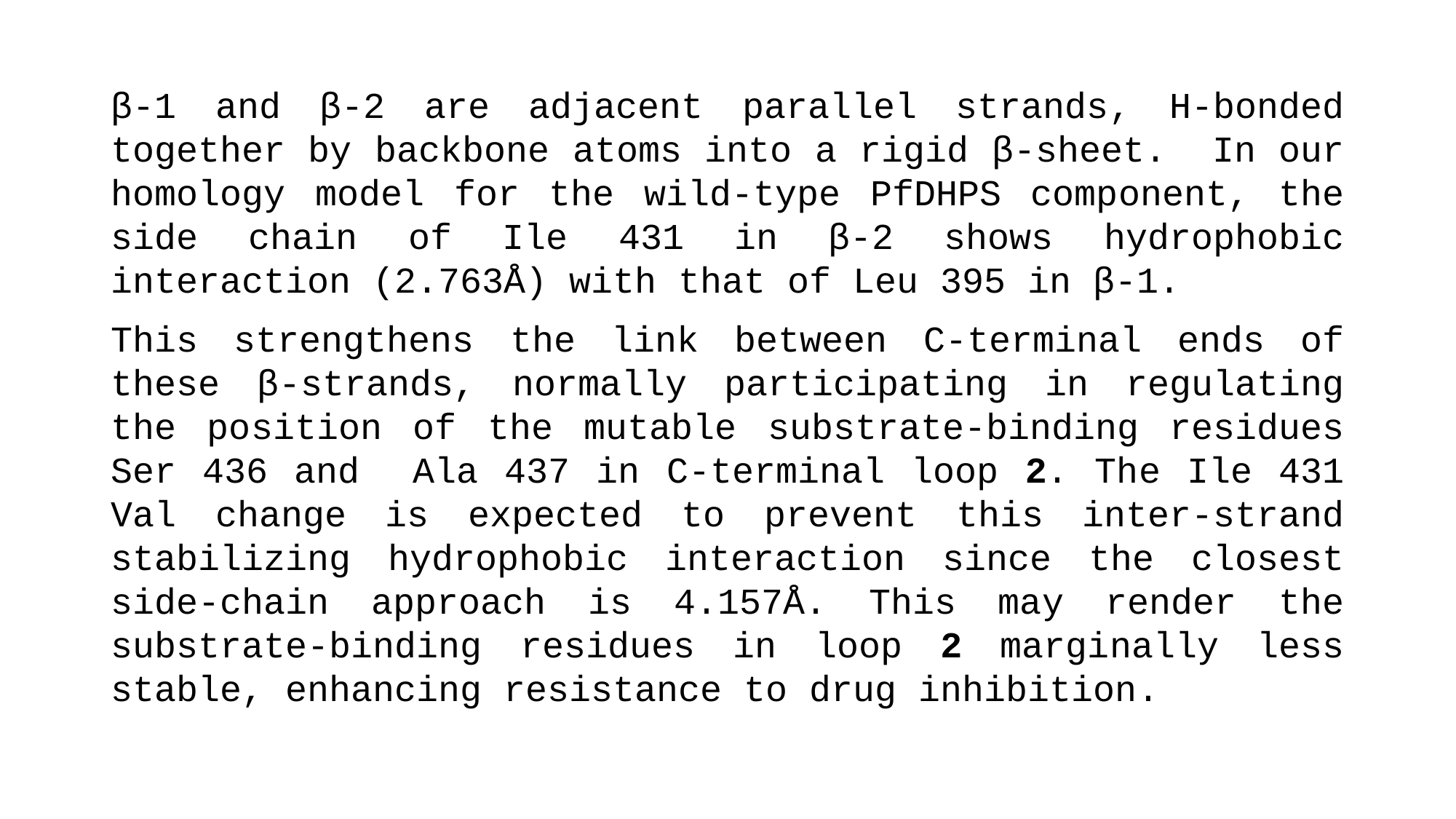

β-1 and β-2 are adjacent parallel strands, H-bonded together by backbone atoms into a rigid β-sheet. In our homology model for the wild-type PfDHPS component, the side chain of Ile 431 in β-2 shows hydrophobic interaction (2.763Å) with that of Leu 395 in β-1.
This strengthens the link between C-terminal ends of these β-strands, normally participating in regulating the position of the mutable substrate-binding residues Ser 436 and Ala 437 in C-terminal loop 2. The Ile 431 Val change is expected to prevent this inter-strand stabilizing hydrophobic interaction since the closest side-chain approach is 4.157Å. This may render the substrate-binding residues in loop 2 marginally less stable, enhancing resistance to drug inhibition.

## Slide 4
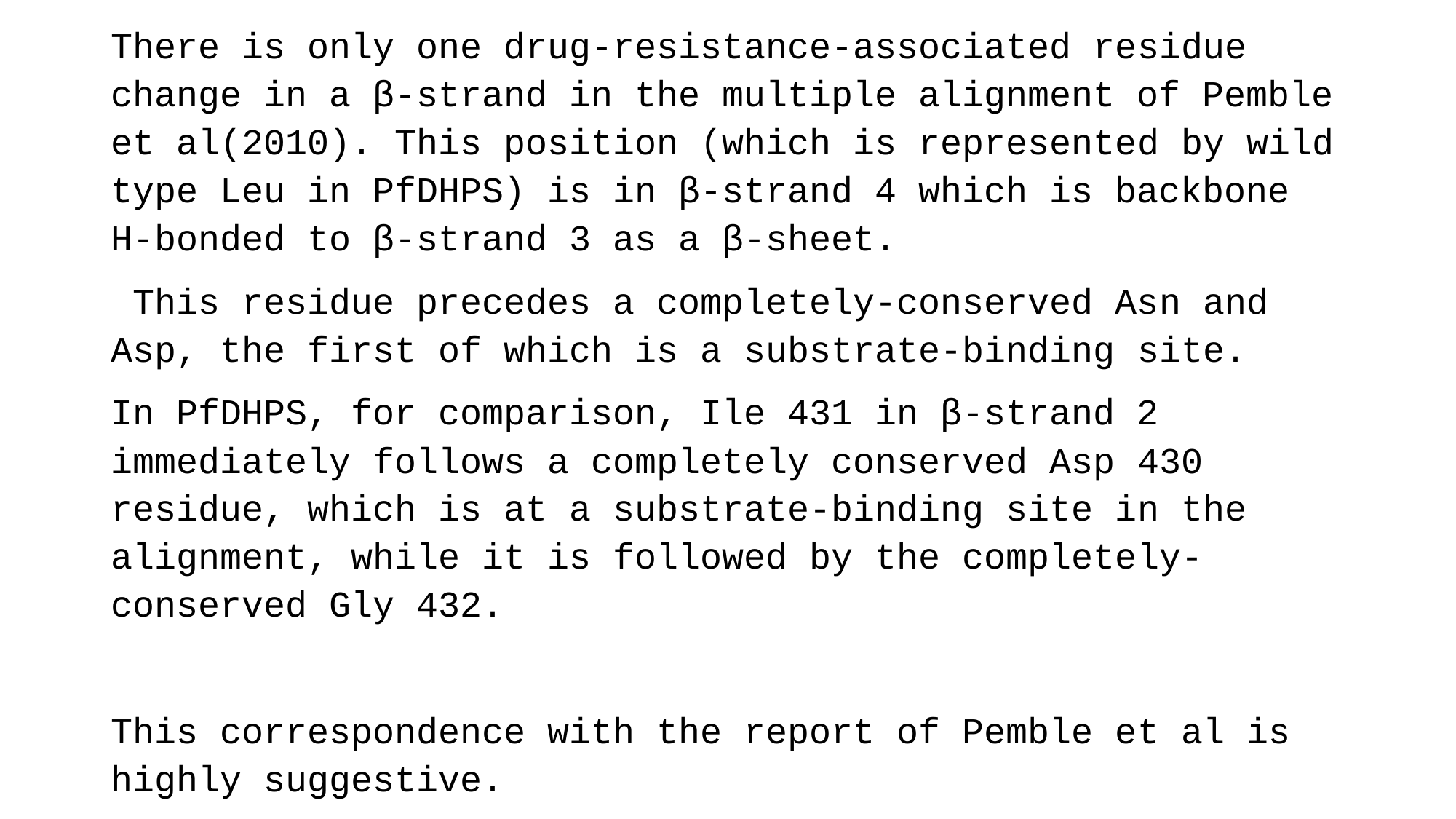

There is only one drug-resistance-associated residue change in a β-strand in the multiple alignment of Pemble et al(2010). This position (which is represented by wild type Leu in PfDHPS) is in β-strand 4 which is backbone H-bonded to β-strand 3 as a β-sheet.
 This residue precedes a completely-conserved Asn and Asp, the first of which is a substrate-binding site.
In PfDHPS, for comparison, Ile 431 in β-strand 2 immediately follows a completely conserved Asp 430 residue, which is at a substrate-binding site in the alignment, while it is followed by the completely-conserved Gly 432.
This correspondence with the report of Pemble et al is highly suggestive.
